# Sputter-Deposited Superconducting Thin Films for Use in SRF Cavities

**DOI:** 10.3390/nano15191522

**Published:** 2025-10-05

**Authors:** Bharath Reddy Lakki Reddy Venkata, Aleksandr Zubtsovskii, Xin Jiang

**Affiliations:** Chair of Surface and Materials Technology, Institute of Materials Science, University of Siegen, 57076 Siegen, Germany; bharath.lakkireddyvenkata@uni-siegen.de

**Keywords:** superconducting radio-frequency (SRF) cavities, superconductors, magnetron sputtering, thin-film coatings, superconductor–insulator–superconductor (SIS) multilayers, accelerating gradients

## Abstract

Particle accelerators are powerful tools in fundamental research, medicine, and industry that provide high-energy beams that can be used to study matter and to enable advanced applications. The state-of-the-art particle accelerators are fundamentally constructed from superconducting radio-frequency (SRF) cavities, which act as resonant structures for the acceleration of charged particles. The performance of such cavities is governed by inherent superconducting material properties such as the transition temperature, critical fields, penetration depth, and other related parameters and material quality. For the last few decades, bulk niobium has been the preferred material for SRF cavities, enabling accelerating gradients on the order of ~50 MV/m; however, its intrinsic limitations, high cost, and complicated manufacturing have motivated the search for alternative strategies. Among these, sputter-deposited superconducting thin films offer a promising route to address these challenges by reducing costs, improving thermal stability, and providing access to numerous high-T_c_ superconductors. This review focuses on progress in sputtered superconducting materials for SRF applications, in particular Nb, NbN, NbTiN, Nb_3_Sn, Nb_3_Al, V_3_Si, Mo–Re, and MgB_2_. We review how deposition process parameters such as deposition pressure, substrate temperature, substrate bias, duty cycle, and reactive gas flow influence film microstructure, stoichiometry, and superconducting properties, and link these to RF performance. High-energy deposition techniques, such as HiPIMS, have enabled the deposition of dense Nb and nitride films with high transition temperatures and low surface resistance. In contrast, sputtering of Nb_3_Sn offers tunable stoichiometry when compared to vapour diffusion. Relatively new material systems, such as Nb_3_Al, V_3_Si, Mo-Re, and MgB_2_, are just a few of the possibilities offered, but challenges with impurity control, interface engineering, and cavity-scale uniformity will remain. We believe that future progress will depend upon energetic sputtering, multilayer architectures, and systematic demonstrations at the cavity scale.

## 1. Introduction

**Figure 1:**
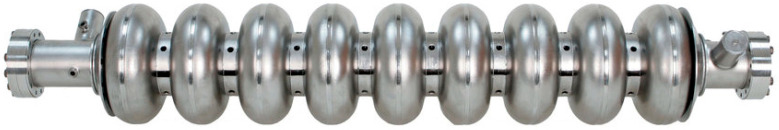
Niobium SRF cavity. Reproduced from the Fermilab SRF Materials Research Department [4].

Superconducting radio-frequency (SRF) cavities are enclosed resonant structures, as shown in Figure 1, used as key components of modern particle accelerators, which transfer electromagnetic energy to charged particle beams, thereby accelerating them to very high speeds with high efficiency. In the early days of particle accelerator development, cavities were made from copper because of its good electrical and thermal conductivity, mechanical robustness, and ease of fabrication [1]. However, when these cavities are operated near room temperature, the electrical resistivity that occurs at that temperature leads to high radio-frequency (RF) losses, and thus very inefficient power transfer from the input RF to the beam. Resistive heating increases quadratically with the RF power, resulting in a loss of efficiency that can lead to high refrigeration demands, reduced quality factors, limiting achievable accelerating gradients, and, in extreme cases, causing structural damage such as cavity melting [2,3]. These limitations pushed the transition to superconducting technology.

Superconducting materials possess surface resistance (R_s_) several orders of magnitude lower than copper, thereby reducing RF power dissipation by factors as high as 10^6^. SRF cavities normally operate at cryogenic temperatures around 2 K and can sustain high duty cycles and even continuous waveforms (CW) operation with accelerating gradients (E_acc_) of ~45 MV/m [5]. Bulk niobium has been the preferred material for SRF cavities for several decades as it has a relatively high critical temperature (T_c_ ≈ 9.2 K), large lower critical field (H_c1_ ≈ 180 mT), and established manufacturing methods. Continuous improvements to bulk Nb processing, including high-purity refining [6], surface treatments [7], and nitrogen doping [8], have pushed cavity gradients close to the intrinsic limit of ~50 MV/m [9,10]. Even with these advances, the limits of Nb’s inherent superconducting properties, moderate thermal conductivity, high production cost, bulk fabrication complexity, and surface processing continue to limit the progress towards higher accelerating gradients [11,12].

Strategies utilising thin-films have emerged as a preferred alternative, due to the very shallow depth of penetration of RF fields in superconductors (for example, ~40 nm for Nb [13] and 200–350 nm for NbN [14]) implies that SRF performance is primarily a surface phenomenon [2]. This has led to the development of the thin-film-coated cavities, as an alternative to bulk Nb structures. In principle, only a micrometre-thick coating is enough to provide the required superconducting properties, while allowing the bulk of the cavity to be made from copper. Such Nb/Cu cavities have been successfully used at LEP-II [15], HIE-ISOLDE [16], and the LHC [17], where they demonstrated both cost and thermal advantages [18]. However, high-field Q-slope, interfacial voids, film adhesion, stress, impurity incorporation remain challenges [19,20,21,22].

In order to achieve higher performance, the research focus shifted to higher-T_c_ superconductors such as NbN (~16 K), NbTiN (~17 K), Nb_3_Sn (~18 K), and MgB_2_ (~39 K), etc. [23]. These materials reduce BCS surface resistance (R_BCS_) and operate more efficiently at 4.2 K; however, they have relatively low H_c1_, and therefore, they may not achieve as high gradients as implemented with Nb [23]. To overcome this limitation, a theoretical framework introduced in 2006 proposed the use of superconductor–insulator–superconductor (SIS) multilayers, in which alternating superconducting and insulating layers are used to shield the underlying superconductor from applied magnetic fields, thereby increasing the maximum accelerating gradient beyond the limits of bulk Nb [24]. Early studies on NbN and NbTiN multilayers deposited on Nb show an increase in H_c1_ and a decrease in R_s_, but there remains the challenge of achieving uniform, defect-free insulating barriers [25,26,27,28].

Numerous reviews have focused on superconductivity [29] and SRF technology [2], with a focus on nitrogen doping [10], bulk niobium, and alternative superconducting materials [23,30]. To date, there has not been a review that has specifically focused on sputter-deposited thin films for SRF cavities. We aim to fill this gap by reviewing sputter-coated superconducting thin films for SRF applications, including Nb, NbN, NbTiN, Nb_3_Sn, Nb_3_Al, V_3_Si, Mo–Re, and MgB_2_. This review will examine the influence of deposition process parameters on the microstructure and superconducting properties of thin films, considering a timeline approach that starts with the earliest studies employing DC magnetron sputtering (DCMS) and extending to recently developed processes like High-power impulse magnetron sputtering (HiPIMS). We will also review achievements, limitations, and remaining challenges, and possible directions for the development of the next generation of SRF cavity coatings. The intended audience will be SRF scientists, thin-film scientists, and the larger community of researchers working on applied superconductivity.

## 2. Superconductivity and SRF Operation

### 2.1. Fundamentals

Superconductivity is characterised by three key phenomena: the complete loss of DC resistance, perfect diamagnetism known as the Meissner effect, and the quantisation of magnetic flux [31]. Zero DC electrical resistance only occurs when a superconductor is cooled below its T_c_, the temperature at which the transition from the normal to the superconducting state occurs, a phenomenon first discovered by Kamerlingh Onnes in 1911 [32]. The Meissner effect [33], discovered in 1933, demonstrated that superconductivity is characterised by the expulsion of magnetic flux. When a superconductor is cooled below its T_c_ in an applied magnetic field, screening currents are induced near the surface that generate a magnetic field equal and opposite to the applied field, thereby forcing flux out of the bulk. This perfect diamagnetism continues to stay until the applied field reaches a sufficient strength to overcome the screening currents, at which point the material returns to the normal conducting state. The field strength at which this transition occurs is known as the critical magnetic field (H_c_). Along with the critical current density (J_c_), these parameters characterise the transition from superconducting to normal conducting behaviour: a material will only display superconductivity when T < T_c_, H < H_c_, and J < J_c_ [31].

**Figure 2:**
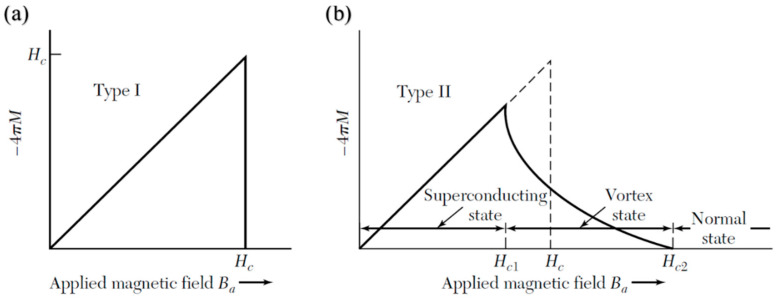
Magnetic response of type I (**a**) and type II (**b**) superconductors showing the critical fields H_c_, H_c1_, and H_c2_. Reproduced from [35].

**Figure 3:**
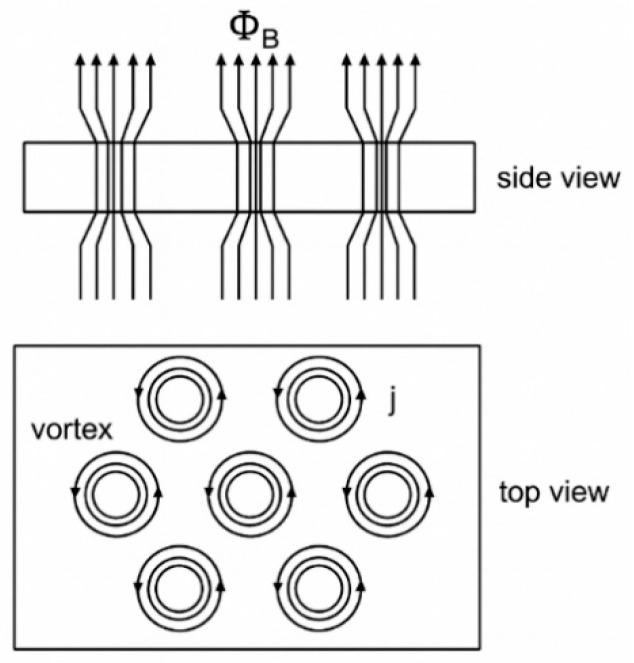
Schematic illustration of magnetic flux vortices penetrating a type II superconductor in the mixed superconducting state. Adapted from [36].

Superconductors are typically classified by their magnetic response. Type I superconductors (Figure 2a) exhibit the Meissner state until H_c_ is reached, above which they abruptly lose superconductivity and enter the normal conducting state, even if the temperature is still below T_c_. In contrast, Type II superconductors (Figure 2b) possess two critical fields. Above the H_c1_, they enter an intermediate or mixed state where magnetic flux enters the material in the form of quantised vortices, each carrying a single flux quantum. This was demonstrated experimentally in 1961 by Deaver and Fairbank [34]. These vortices consist of normal-conducting vortex cores surrounded by circulating supercurrents that confine the magnetic field locally, allowing the material to maintain zero DC resistance, as shown in Figure 3, and they are usually pinned at defects or impurities within the material. As the applied field increases, the number of vortices grows until they overlap at the upper critical field (H_c2_), beyond which superconductivity is destroyed. A very stable mixed states make Type II superconductors a very valuable material for high-field applications, including SRF cavities [31].

### 2.2. Superconductivity Theory

Gorter and Casimir [37] were the first to describe superconductivity using a two-fluid theory. They describe that, below T_c_, a fraction of the electrons condense into a zero-resistance state called superfluid, while the rest remain in the normal state. As the temperature decreases, the superfluid fraction increases, allowing lossless current to flow. Superelectrons carry current without loss under a DC field; however, under an AC field, their inertia prevents them from mimicking the oscillating field instantaneously, and the residual normal electron fluid accelerates and creates a resistance effect. Expanding on this idea, Fritz and Heinz London (1935) [38] formulated the London equations, which were able to explain the Meissner effect by introducing the concept of the London penetration depth (λ_L_). This parameter is a measure of the characteristic length (decay length) of an externally applied magnetic field inside a superconductor, and beyond this length, the material is in the Meissner state. The penetration depth is a material property that is dependent on the density, mass, and charge of the superelectrons.

In 1950, Ginzburg and Landau’s [39] development of GL theory is a thermodynamic description of superconductivity near T_c_, which describes changes in the superelectron density at normal—superconducting boundaries. They introduced the complex order parameter (Ψ), which is zero in the normal state and greater than zero in the superconducting state, and the superelectron density is defined as n_s_ = |Ψ|^2^. Based on this, the Ginzburg–Landau coherence length (ξ_GL_) is defined, which is the characteristic distance over which the order parameter changes significantly, and the penetration depth (λ_GL_), which has the same physical meaning as the London penetration depth (λ_L_). Collectively, these two quantities form the Ginzburg–Landau parameter (κ = λ_GL_/ξ_GL_) to characterise the magnetic response of the material at the interfaces. κ < 1/√2 corresponds to type I, while κ ≥ 1/√2 corresponds to type II.

In 1957, Bardeen, Cooper, and Schrieffer introduced the BCS theory [40], which offered the first microscopic model of superconductivity. While it has its limits of applicability, the BCS theory is still the most successful and most widely employed model for conventional superconductors. The theory describes the formation of Cooper pairs below T_c_ when the phonon-mediated attraction between electrons overcomes thermal agitation. Theoretically, when an electron travels through a metal, its Coulomb attraction to positively charged ion cores locally distorts the lattice. This distortion produces a region with a net positive charge, which attracts another electron in the superconductor. This interaction is called Cooper pairing. Cooper pairing lowers the electronic energy near the Fermi surface and gives rise to the superconducting state. A major prediction of BCS theory is the existence of an energy gap (Δ) between the superconducting and normal states. This energy gap corresponds to the binding energy of Cooper pairs and to break a Cooper pair requires an energy to 2Δ. The energy gap decreases with increasing temperature and essentially goes to zero at T_c_; this explains the absence of superconductivity at the transition.

### 2.3. Surface Resistance in RF Fields

While superconductors possess zero electrical DC resistance, they are observed to have a finite R_s_ with alternating electromagnetic fields, as is the case with RF use. Above absolute zero, not all electrons are Cooper pairs; some remain as normal electrons. Under a DC, Cooper pairs can flow with no resistance, but under an RF current, Cooper pairs must always reverse their direction of travel. The Cooper pairs have inertia, and thus when they are accelerated and decelerated, a small electric field is induced in the London penetration depth (λ_L_), where supercurrents flow. Moreover, this electric field induces motion in the remaining normal electrons, causing them to absorb energy from the RF field, and this energy dissipates as heat. The temperature-dependent contribution is described by BCS theory and expressed as the BCS surface resistance ($R_{BCS}$, and is given by [40]:

$$R_{BCS}\;\left(f,\;T\right)=A\frac{f^{2}}{T}\exp\left(-\frac{∆}{K_{B}T}\right)$$

where Δ is the superconducting energy gap, *f* is the RF, *T* is the temperature, and *A* is a material-dependent constant. The constant *A* incorporates superconductor properties such as the London penetration depth (λ_L_), electron mean free path (l), coherence length (ξ_0_), and normal-state resistivity, meaning that its value differs from one superconductor to another.

With T = 0 K, R_BCS_ should, in theory, vanish. However, in practice, there will always be a residual surface resistance (R_res_) that remains. R_res_ is temperature independent, and the BCS theory does not take it into account. R_res_ can originate from a variety of extrinsic influences, including trapped magnetic flux, impurities larger than the coherence length, grain boundaries, oxides, chemical residues, hydride formation, surface roughness, and gas inclusions from the deposition process [41]. Interestingly, studies have shown that R_res_ is often proportional to the square root of the normal-state resistivity (√ρ_n_) [23], highlighting the influence of normal-state material properties on RF performance. In addition, trapped flux causes fluxon-induced resistance (R_fl_). When a cavity cools through T_c_, a portion of the external magnetic field is trapped at pinning sites, such as structural defects (grain boundaries and inclusions), which might be created during the fabrication of the cavity. The trapped vortices will oscillate and dissipate energy under RF fields, thus causing more losses. While strong pinning can typically immobilise the vortices and thus reduce losses, weak or partial pinning allows vortices to move in RF fields, drastically lowering performance. Therefore, minimising R_res_ and R_fl_, through magnetic shielding, careful cooldown protocols, and control of impurities and surface defects, is very important for maximising the intrinsic quality factor (Q_0_) of SRF cavities.

## 3. SRF Cavity Performance: Figures of Merit and Material Strategies

For SRF applications, the geometry of the cavity and the operating frequency are functions of the particle velocity (β = v/c), with quarter-wave or half-wave designs used for low-β beams and elliptical TM-mode cavities used for high-β beams. Two figures of merit define their performance: the accelerating gradient (*Eacc*) and the intrinsic quality factor (*Q*_0_). The accelerating gradient is defined as

$$E_{acc}=\frac{V_{acc}}{z}$$

where *V_acc_* is the accelerating voltage and *z* is the cavity length. The quality factor relates the stored energy to the power that dissipates:

$$Q_{0}=\frac{\omega U}{P_{d}}=\frac{G}{R_{s}}$$

where ω is the angular frequency, U the stored energy, $P_{d}$ the dissipated power, *G* the geometry constant, and $R_{s}$ the surface resistance. With high *E_acc_*, the number of cavities required in an accelerator is reduced, and with high *Q*_0_, refrigeration load and operational costs are minimised.

Nb remains the most widely used material for SRF applications, possessing the highest superconducting transition temperature (T_c_ ≈ 9.2 K) among the elements, relatively high lower critical field (H_c1_ ≈ 180 mT at 2 K), and well-established fabrication techniques. Available methods of surface processing and preparation throughout the years, including electropolishing [9], high-pressure rinsing [42], 120 °C baking [43], and nitrogen doping [10], have allowed bulk Nb cavities to reach accelerating gradients near 50 MV/m, which closely approaches the theoretical limits of Nb. Throughout that process, performance-limiting issues like hydrogen precipitation (Q-disease), multipacting, thermal quenches, and field emission have been observed and systematically improved. That said, bulk Nb cavities are costly to manufacture, rely heavily on chemical treatments that may cause hazardous waste, and rely on Nb’s relatively poor thermal conductivity, indicating a need for alternatives. One established route is utilising thin-film superconductors on a copper substrate, with micrometre-scale coatings of Nb providing the superconducting properties and Cu cavities providing mechanical stability and high thermal conductivity. Nb/Cu technology has been successfully implemented in facilities such as LEP-II and the LHC, allowing for a lower cost of materials, higher thermal stability, and lower sensitivity to trapped magnetic flux. However, there are still limitations, the most significant being the Q-slope that occurs at higher fields, as well as the sensitivity to substrate preparation, interfacial voids, and incorporation of impurities during the sputtering process. Research is therefore focused on new energetic deposition methods in a forthcoming chapter.

Besides Nb, other superconductors with higher T_c_, specifically Nb_3_Sn (T_c_ ≈ 18 K), NbN (T_c_ ≈ 16 K), and NbTiN (T_c_ ≈ 17 K), offer an opportunity to decrease BCS surface resistance and thereby increase the overall efficiency for operation at 4.2 K. Other candidates may include V_3_Si, Nb_3_Al, and MgB_2_. The specific superconducting parameters of interest are T_c_, H_c_, H_c1_, H_c2_, penetration depth (λ), superconducting gap (Δ), and coherence length (ξ), summarised in Table 1. These data [23] present a positive basis for moving beyond Nb.

**Figure 4:**
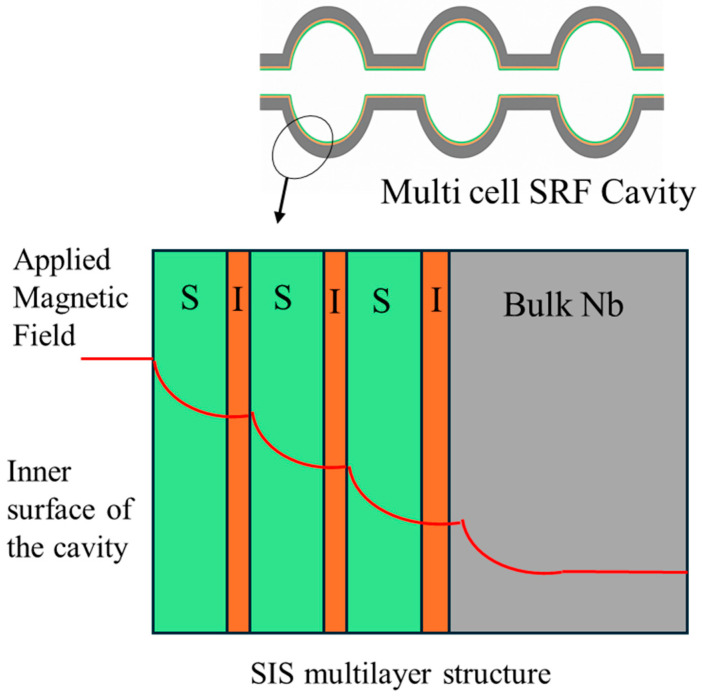
Schematic representation of the Superconductor–Insulator–Superconductor (SIS) multilayer concept applied to a multi-cell SRF cavity. Adapted from [45].

One potential mechanism to improve the performance of superconductor materials beyond the bulk limits of Nb is through a Superconductor–Insulator–Superconductor (SIS) multilayer system, first proposed by Gurevich. In an SIS structure, the Nb substrate is coated with alternating thin insulating (I) and superconducting (S) layers; the outer S layer has a higher T_c_ and larger superconducting gap, such as Nb_3_Sn, NbN or NbTiN. Advantages of a multilayer structure include improved magnetic screening and less RF surface resistance: thin superconducting layers with thicknesses less than their penetration depth (λ) suppress flux entry and increase the effective superheating field, allowing operation beyond Nb’s intrinsic limit of ~200 mT. Additionally, these high-T_c_ superconductors reduce the BCS surface resistance, especially at 4.2 K, allowing for larger cryogenic savings. Insulating layers decouple neighbouring superconducting films, reduce the Josephson coupling, and can limit the vortex propagation across the multilayer stack, thereby delaying the magnetic flux penetration into the Nb substrate. Multilayers of NbN and NbTiN, which are deposited on a Nb substrate, have demonstrated enhanced H_c1_ and lower surface resistances than bulk Nb. The main challenge is to develop an effective insulating layer (AlN or MgO) that is uniform, dense, and defect-free, which can effectively decouple the superconducting films without forming weak links and pinholes. Other dielectrics such as SiO_2_ and Al_2_O_3_ can also be used as insulating layers; however, most recent studies focus on AlN and MgO, which usually provide more uniform barriers. This preference may be related to pinholes or weak spots that have been reported in some oxide barriers [44]. A schematic of the SIS idea is shown in Figure 4.

## 4. Sputtering Techniques for SRF Thin-Film Coatings

In SRF research, sputtering has become the dominant approach to deposit superconducting thin films, beginning with Nb/Cu technology and then to other compounds such as NbN, NbTiN, and Nb_3_Sn, etc. In contrast to bulk fabrication routes or diffusion-based coatings, sputtering allows for good control of deposition on non-planar geometries in a scalable way. There are several parameters of the deposition process that can be tuned during the film deposition. Film density, crystallinity, degree of adhesion, and stoichiometry are influenced either directly or indirectly by the plasma characteristics and deposition conditions. Such properties and conditions influence superconducting properties relevant to SRF. This chapter summarises the traditional sputtering techniques found in the literature from the earliest diode configurations to magnetron-based processes, reactive processes, pulsed DC, and finally, modern HiPIMS, discussing the mechanisms through which they operate and the advantages and disadvantages, with attention to their effects on film growth and suitability for RF cavity coatings.

### 4.1. Principles of Plasma Generation in Sputtering

**Figure 5:**
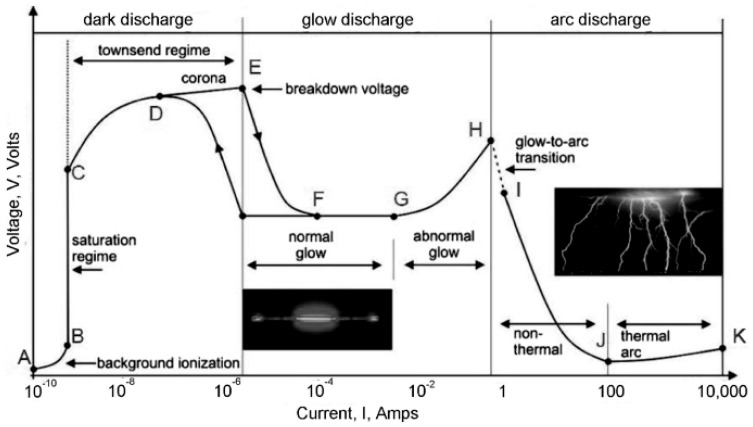
Distinct regimes of plasma discharge. Reproduced from [46].

The generation of plasma is the primary step in sputtering, which can be interpreted via the voltage–current characteristics of electrical discharges, as shown in Figure 5. Sputtering begins with the introduction of a neutral process gas, such as argon or krypton, into the chamber. Once an electric field is applied, free electrons are accelerated, leading to collisions that ionise the gas (Townsend discharge). These ions accelerate towards the cathode surface and bombard it, ejecting target atoms and releasing secondary electrons that sustain the discharge. Once the breakdown takes place, the plasma establishes in the normal glow regime, a stable plasma condition where processes yield a visible light emission due to electron–ion recombination. As the electrical potential (applied field) is increased, the discharge enters the abnormal glow regime where carrier densities (10^15^–10^19^ m^−3^) are sufficiently high to sustain sputtering. On further increasing the field, the discharge transitions into arc mode, where significant current densities and target material degradation occur, as this condition is unsuitable for thin-film deposition.

For SRF applications, it is essential that the processes operate in the abnormal glow regime, since stable ion fluxes dictate the uniformity, density and stoichiometry of the superconducting coating. Fluctuations in the plasma conditions directly translate to non-uniform film growth, impurity incorporation, or defects in film microstructure that impede the superconducting properties. Thus, subsequent sputtering techniques developed largely from this basic principle and used magnetic confinement or pulsed power to maintain a stable discharge while increasing the ionisation efficiency.

In the sputtering process, atoms from a solid target are sputtered away by bombarding ions created in a plasma environment. When ions strike the target surface, they may reflect, be incorporated into the lattice, or cause collision cascades leading to the ejection of target atoms. The ejected species travel through the plasma and condense on the surface of the substrate, forming a thin film. Sputtering occurs only when the energy of the incident ions exceeds the threshold energy needed to overcome the forces that bind atoms together, and this threshold energy is a function of several properties, such as the mass ratio between the ion and target atoms, surface binding energy, and the angle of incidence of the ions. Sputtering yield, the number of atoms ejected per incident ion, measures the efficiency of material removal and generally increases with cathode voltage, and is maximised when the mass of the process gas is close to the mass of the target atoms. The energy of ejected atoms is usually of a few eV to tens of eV and described by a Thomson-type distribution [47,48].

In SRF applications, these sputtering mechanisms are directly related to the quality of the coating. For instance, poorer superconducting quality is associated with low-energy sputtered species or excessive scattering at high deposition pressures, resulting in porous, columnar films. Ion bombardment control can increase adatom mobility, and as a result, more dense coatings are formed, which adhere well and have better T_c_ values with lower RF surface resistance. The most critical parameters are deposition pressure, cathode voltage, substrate bias and temperature, and process gas selection. These parameters will play a significant role in achieving the trade-off between the density, stoichiometry, and stress of the film. There are several sputtering methodologies that have been developed to try to balance these competing requirements, including diode, RF, magnetron, (HiPIMS), etc. However, magnetron-based methods have been the most commonly utilised approach in the study of SRF cavities because they are able to deposit dense, uniform superconducting films on more complicated geometries [49,50,51].

### 4.2. DC Diode Sputtering

The DC diode system was the first sputtering method introduced. It consists of a very simple configuration with a cathode and an anode. This is typically operated at chamber pressures of 1–100 mTorr and voltages of 2–5 kV, in which Ar^+^ ions bombard the negatively biased cathode and eject target atoms that coat the substrate. This is a simple and cost-effective method; however, it is only feasible with conducting targets and is prone to arcing with insulators. For SRF applications, films deposited by DC diode sputtering were shown to contain voids, weak adhesion, and high levels of incorporated gas, leading to suppressed T_c_ and high residual resistance [52,53]. This technique was quickly replaced by magnetron-based techniques, which provide denser and more uniform coatings [47,54].

### 4.3. RF Diode Sputtering

RF diode sputtering was developed to address the undesirable charge-accumulation problem associated with DC sputtering when using insulating targets. An RF power supply is capacitively coupled to the cathode. The resulting effect is that the target potential switches between positive and negative. In the negative half-cycle of the RF wave, energetic ions strike the target surface to eject atoms, while in the positive half-cycle, the accumulated charge is neutralised by electrons. Because electrons respond to changes more quickly than ions, a net negative self-bias develops on the target, maintaining a constant ion bombardment that affects film density and adhesion. RF sputtering has the advantage of being able to operate at much lower pressures (<1 mTorr) and can be applied to both conducting and insulating targets. However, RF sputtering has a lower deposition rate than DC sputtering, and high power levels can induce thermal stress in brittle targets. For SRF materials, this limitation is compounded by the fact that RF diode sputtering often produces low-density films with limited control over microstructure, which does not meet the requirements for high-quality superconducting coatings [55]. Its utility is mostly as experimental evidence for an alternative method for coating insulating layers. For metallic superconductors like Nb, NbN, or NbTiN, magnetron-based methods have proven much more suitable [47,56].

### 4.4. Magnetron Sputtering

**Figure 6:**
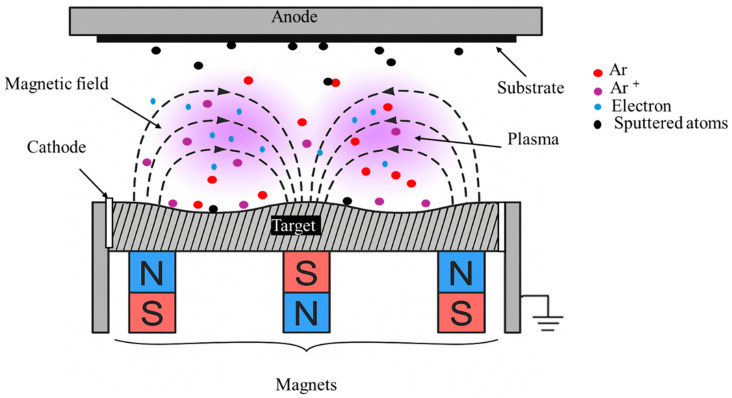
Schematic of a planar magnetron sputtering system. A magnet array behind the target confines the plasma in a racetrack zone. Adapted from [59].

Traditional DC sputtering operates at high voltages (2–5 kV) and relatively high pressures, which significantly decrease deposition rates, limit the mean free path of the sputtered atoms, and promote gas incorporation into the thin film as it grows. Magnetron sputtering improves upon these issues by placing permanent magnets behind the cathode to make crossed electric and magnetic fields (E × B) that effectively trap electrons in close proximity to the target surface (Figure 6). Longer electron paths improve the efficiency of ionisation and cause a racetrack-shaped plasma above the target. The confinement of plasma increases the sputtering yield and deposition rate, while minimising sputtering of the chamber walls. On the downside, it also results in preferential erosion of the target along the magnetic field lines and therefore creates the racetrack wear pattern.

Magnetron sputtering operates at lower voltages (~300 V) and pressures (<5 mTorr) leading to films with higher density and fewer gas defects than diode sputtering. Planar magnetrons are typically used for flat substrates, whereas cylindrical geometries enable the uniform coating of the curved inner surface of SRF cavities. Depending on the type of power supply, i.e., [57]. DC, pulsed DC or RF, different plasma conditions can be established. Out of these options, DC magnetron sputtering (DCMS) became the most widely used technique for SRF studies, allowing the deposition of Nb and compound superconductors in a scalable way. Nb/Cu technology used at LEP-II and the LHC was based on DCMS [58]. However, due to the low energy of sputtered species, deposited films are typically porous and columnar in structure with poor adhesion, which leads to high-field Q-slope and high residual surface resistivity. These drawbacks motivated the development of more energetic variants, most notably HiPIMS [47,57].

### 4.5. Reactive Sputtering

Reactive sputtering is a commonly used technique to deposit compound films with controlled stoichiometry, most notably nitride and oxide films whose bulk forms cannot be fabricated. This process involves the reactive sputtering of an elemental or alloy target in an atmosphere of a reactive gas (e.g., N_2_, O_2_) and the introduction of process gas as an inert gas. The reactive species not only reacts with the sputtered atoms at the substrate, but it forms a compound layer on the target surface as well. Once this layer forms, known as target poisoning, there is a reduction in the sputtering yield. The quality of the film will depend on how these poisoned states are controlled. Operating in the fully poisoned regime provides stable but low-density films, while operating in the transition zone provides higher quality and stoichiometric coatings with a significant reduction in stability. A predominant obstacle is the hysteresis effect. After reducing the flow of reactive gas, the target does not instantaneously revert to its metallic mode, resulting in unstable conditions and, therefore, non-uniform films. The degree of hysteresis depends on the target composition, gas type, pumping speed, and overall geometry of the system. Reactive sputtering has so far been the primary technique to deposit superconducting nitrides such as NbN and NbTiN for SRF applications. Appropriate stoichiometry is important for these sputtered films, since films that are either N-poor or N-rich have strongly suppressed T_c_ as well as higher surface resistance [47].

### 4.6. Pulsed DC Magnetron Sputtering

Pulsed DC magnetron sputtering was developed primarily for the purposes of optimising the reactive sputtering of dielectric and compound films, in which the application of continuous DC bias contributes to charge build-up and induces arcing. In this technique, the applied voltage alternates between negative and positive pulses that discharge the target surface more quickly, while sustaining the plasma during the sputtering process. The alternating polarity eliminates arcing, enhances plasma stability, and enables higher current densities than DCMS, thereby enhancing film density for conductive materials. In addition, pulsed DC offers a less complex power supply than RF diode sputtering and can be successfully utilised to deposit dielectric or compound films. In the magnetron configuration, along with the pulsing capabilities, the high plasma density enhances control over stoichiometry in reactive processes. Specifically, with respect to SRF applications, pulsed DC magnetron sputtering offers a reliable route to the deposition of NbN and NbTiN coatings, without the hysteresis and instabilities found in conventional reactive sputtering. According to studies that have been reported, this stabilisation provides denser films and sharper superconducting transitions, with pulsed DC representing an important intermediate option between conventional DCMS and more advanced energetic techniques, e.g., HiPIMS [60,61,62].

### 4.7. High Power Impulse Magnetron Sputtering (HiPIMS)

HiPIMS is an advanced variant of magnetron sputtering with magnetic confinement and high-power pulsed operation. In HiPIMS, during each pulse, a significant amount of stored energy is released in microseconds, producing plasma densities that can be orders of magnitude greater than those in DCMS. A large proportion of sputtered atoms will be ionised, and the trajectories of the ions can be redirected by substrate bias. This form of deposition provides higher adatom mobility and produces a film with high density, smooth surface, and good adhesion. Additionally, self-sputtering (ionised species circling back to the target to eject additional atoms) also helps sustain the discharge more. The instantaneous power of HiPIMS is very high. At the same time, the pulsed nature of HiPIMS provides a very low time-averaged power, which helps to reduce the possibility of overheating the target [63].

**Figure 7:**
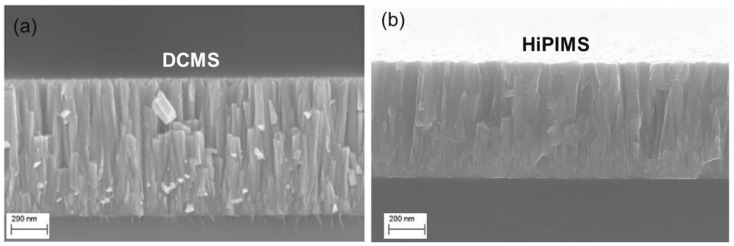
Cross-sectional SEM comparison of NbTiN films deposited by (**a**) DCMS and (**b**) HiPIMS. Reproduced from [65].

The HiPIMS films are demonstrated to have far greater density and crystallinity than the DCMS films, with much lower void content and superior adhesion. The main concern with HiPIMS is the lower deposition rate, especially at low duty cycles, as well as the greater complexity of the system. However, there are some applications, particularly superconducting coatings like Nb, NbN, and NbTiN, where HiPIMS performance has been extremely useful, especially where dense, defect-free coatings with bulk-like lattice parameters are needed to minimise RF losses and suppress the Q-slope. The benefits of HiPIMS technology are illustrated in Figure 7, which compares the NbTiN thin films deposited at the same pressure (1.8 Pa) by DCMS and HiPIMS. The morphology of the DCMS film is porous, columnar, and features internal voids, with a relatively low T_c_ (~11.8 K). HiPIMS film, on the other hand, was dense and uniform and provided a distinctly higher T_c_ (~13.7 K), which directly relates microstructure to superconducting performance. This shows the advantage of HiPIMS to potentially mitigate the limitations of traditional sputtering for SRF cavity coatings [47,49,64].

Building on these advances in deposition methods, the following chapter examines how different superconducting materials have been realised through sputtering, with an emphasis on the relationship between deposition conditions, microstructure, and RF performance.

## 5. Sputtered Superconducting Materials for SRF Cavities

Having surveyed the various techniques for sputtering, we will now shift our focus to the application of sputtering techniques to deposit different superconducting thin films for SRF cavities. The choice of material is critical due to the influence of T_c_, H_c_, H_c1_, H_c2_, microstructure, and R_s_ on cavity performance concerning achievable intrinsic quality factors and accelerating gradients. Nb continues to be the benchmark material, but alternative materials, such as NbN, NbTiN, Nb_3_Sn, Nb_3_Al, V_3_Si, Mo–Re, and MgB_2_, have also been investigated due to their high T_c_. We will review progress in these material systems with emphasis on the effects of deposition conditions on superconductivity, structure, and RF performance.

### 5.1. Niobium (Nb)

**Figure 8:**
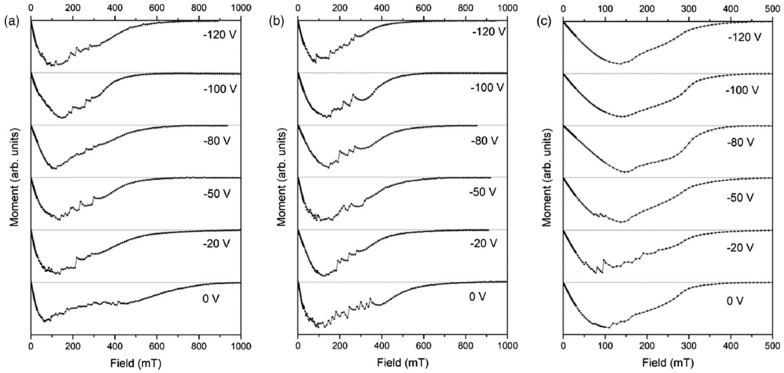
DC magnetic moment versus applied magnetic field at 4.2 K for Nb thin films deposited at (**a**) room temperature, (**b**) 500 °C, and (**c**) 700 °C under different substrate biases. Reproduced from [67].

**Figure 9:**
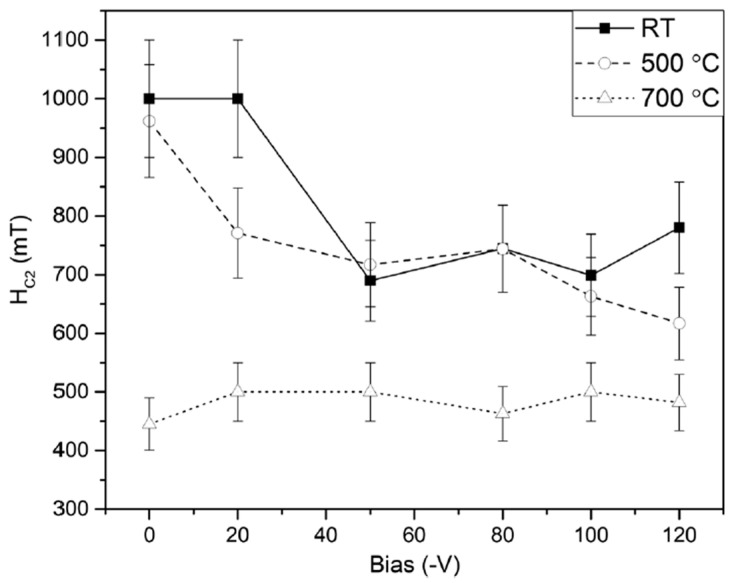
Upper critical field (H_c2_) of Nb thin films as a function of substrate temperature and applied bias. Reproduced from [67].

Nb continues to be the preferred choice for SRF cavities. Its relatively high T_c_ (~9.2 K) compared to other elements and the highest lower critical field (H_c1_ ≈ 180 mT at 0 K) have enabled the development of bulk Nb cavities that now operate very close to intrinsic material limits. Nb has an RF penetration depth of about 40 nm, making Nb thin films on Cu attractive because it combines the benefits of Nb superconducting performance with the thermal conductivity, mechanical strength and cost savings of the Cu. Early studies of Nb thin films showed the critical effect of substrate temperature on the microstructure and superconducting properties of Nb thin films. In DCMS experiments in ultra-high vacuum conditions (2-inch Nb target, Ar, 1 mTorr), Nb/Cu films deposited at a substrate temperature of 150 °C exhibited coarser grains, sharper superconducting transitions, and doubled H_c1_ (10 mT) when compared to films deposited at room temperature (5 mT), while retaining T_c_ ≈ 9.2 K [66]. This observation, that a very small variation in substrate temperature could affect the quality of the film, became a guiding principle for future work. With the availability of energetic deposition techniques, researchers sought to investigate this temperature sensitivity further. HiPIMS was used to deposit Nb films, systematically varying the temperature of the substrate between ambient and 650 °C. Cavity tests at 7.8 GHz showed that the room temperature films exhibited very high RF losses (R_s_ ≈ 181 µΩ at 4.2 K), while the films deposited around 500–550 °C exhibited R_s_ that was very close to the theoretical BCS limit (≈20 µΩ) with T_c_ ≈ 9.1 K [66]. Substrate heating was not only a benefit but also a necessity for producing high-quality Nb coatings. The overall impact of the substrate bias and temperature was subsequently explored using HiPIMS in krypton plasma. The films deposited at 700 °C and with a moderate negative bias (−0 to −120 V) produced smooth magnetisation curves (i.e., no flux jumps and stable flux pinning) (Figure 8). These Nb films exhibited upper critical fields (400–500 mT) that were lower than the fields of the Nb films deposited at room temperature and 500 °C (~700–1000 mT), as can be seen in Figure 9, but these Nb films were much more reproducible and stable in the Meissner state [67]. This interesting trade-off, within the context of having a higher H_c2_ at low temperatures and improving flux stability at elevated temperatures, clearly highlights the competing interactions involved in optimising Nb films for cavity applications.

**Figure 10:**
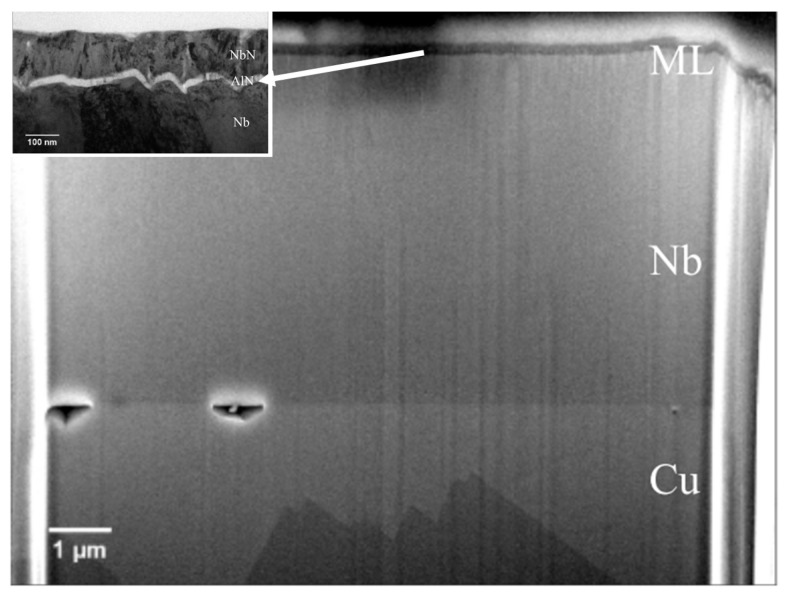
SEM image showing a FIB cut through the full thickness of the SIS structure. Reproduced from [69].

In a comprehensive parameter study systematically varying bias, duty cycle and temperature during the HiPIMS depositions on electropolished copper, it was confirmed that the films with lattice parameters similar to that of the bulk and with large crystallite sizes have the highest T_c_ (~9.3 K average, up to peaks of 9.6–9.7 K) when applying high substrate bias (≥−150 V) and relatively high substrate temperatures (~290 °C) [68]. In another related study, the same authors developed multilayer Nb/AlN/NbN SIS structures, with the Nb base layers achieving T_c_ ≈ 9.4 K and H_en_ ≈ 52 mT alone, with multilayers providing a further improvement to H_en_ of 64.5 mT. From the FIB–SEM cross sections (Figure 10), it can be seen that interfacial voids and partial delamination at the Cu/Nb interface, indicating that even with SIS multilayers, the quality of the Nb base layer can still limit performance [69].

Besides the influences described above for bias and temperature, further microstructural investigations revealed the influence of process pressure, angle, and substrate structuring. Rao et al. showed that Nb films sputtered using DCMS on Si at low pressure (0.15 Pa) were dense, smooth, and compressive with low resistivity (~30 µΩ·cm). In contrast, higher pressures (0.60 Pa) yielded porous, tensile films with high resistivity (~375 µΩ·cm). GIXRD showed that an increase in deposition pressure correlates with an increase in lattice strain, and a decrease in crystallite size, consistent with film degradation at high pressure [70]. In subsequent work, the same group reported that deposition at oblique angles (up to 50°) progressively increased surface roughness (0.4 to 1.5 nm) and resistivity (79 to 293 µΩ·cm), while stresses shifted from compressive to tensile [71]. It was also shown that a very strong negative bias (−300 V) during HiPIMS on trenched Si substrates promoted ion bombardment and re-sputtering, effectively suppressing self-shadowing and producing dense, planarized Nb films, showing that strong ion bombardment during HiPIMS enables uniform deposition on structured substrates [72]. This ability for uniform deposition on structured substrates is critical for coating the complicated geometries of SRF cavities.

**Figure 11:**
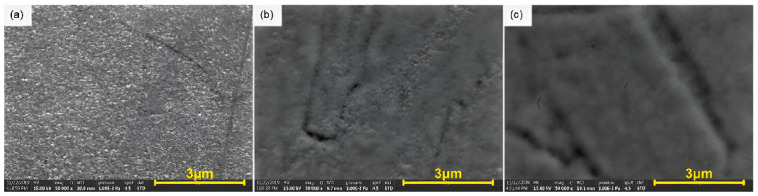
Environmental Scanning Electron Microscopy (ESEM) images of Nb/Cu films deposited at 450 °C by DC magnetron sputtering: (**a**) as-deposited (**b**) after laser annealing at 1.30 J/cm^2^ (one cycle), and (**c**) after laser annealing at 1.30 J/cm^2^ (five cycles). Reproduced from [73].

The deposition process alone cannot solve all issues; therefore, post-deposition treatments were investigated. It has been shown that the nanosecond pulsed laser annealing of DC-sputtered Nb/Cu films at 450–650 °C modified the surface morphology. As shown in Figure 11, a single irradiation laser cycle smoothed granular films, while five cycles reduced surface roughness by approximately 75%. Increased structural refinement was correlated with reduced oxygen/hydrogen contamination and improved connectivity of grains, while all the films exhibited a T_c_ of ~9.3 K. This study showed that laser annealing can effectively reduce surface roughness and Q-slope-related defects [73].

**Figure 12:**
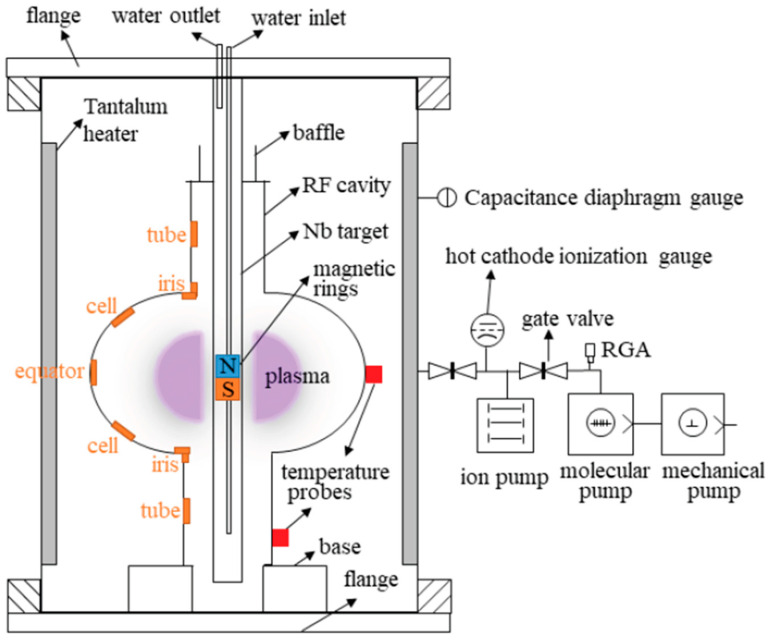
Schematic of the HiPIMS deposition system for coating 1.3 GHz Cu cavities. Reproduced from Duan et al. [74].

This progress encouraged cavity-scale demonstrations. HiPIMS was used to deposit ~1 µm Nb films on Cu substrates and SRF cavities. The films grown on Cu substrates displayed bulk-like lattice parameters, smooth, dense microstructures, and a T_c_ of ~9.5 K. The first cavity coatings achieved Q values in the mid-to-high 10^9^ range up to ~10 MV/m, limited by field emission. Following the high-pressure water rinse and the optimisation of the cathode positioning, accelerating gradients of ~21 MV/m were achieved without field emission, demonstrating that HiPIMS can produce bulk-like Nb films [42]. To further investigate coating uniformity inside cavities, a custom HiPIMS deposition system was developed, which consisted of a coaxial cylindrical magnetron with a movable magnetic ring and a cylindrical Nb target, to coat a 1.3 GHz dummy cavity (Figure 12). Nb films deposited in Kr plasma (0.6 Pa, 490 V, 110 A, 200 °C, 6 h) displayed dense, bulk-like grains (300–800 nm) with RRR ≈ 33. The lattice parameters are close to those of bulk Nb. While all the coatings at the equator were dense and well-adhered, nanoscale porosity was observed in the vicinity of the iris. The T_c_ was constant at 9.24–9.27 K, with a narrow ΔT ≈ 0.2 K, this provides further evidence that HiPIMS can produce high-quality cavity coatings. The presence of nanoscale porosity at the iris suggests that careful optimisation of the deposition parameters is still required to create fully uniform films and ensure reliable suppression of the Q-slope in Nb thin-film cavities [74].

Finally, efforts have been made to further improve cavity performance using annealing approaches. It has been shown that in situ annealing of HiPIMS Nb films at 340 °C improved the quench field from 10.0 to 12.5 MV/m. Furnace annealing at 600 °C and 800 °C further increased the quench field to 13.5 and 15.3 MV/m, respectively. Prolonged annealing at 800 °C (6 h) pushed the quench field to 17.5 MV/m. However, annealing at 900 °C caused Q-switching. This shows that controlled annealing treatments can reduce the medium-field Q-slope, while excessive heating can introduce new instabilities [75]. The results achieved with Nb thin films (~21 MV/m with Q ≈ 10^9^) [42] are promising, although not at the performance level of state-of-the-art bulk Nb cavities (~50 MV/m with Q ≈ 10^10^) [76]. Other issues, including interfacial voids, film uniformity and medium-field Q-slope suppression, suggest Nb thin films are still an area of research.

### 5.2. Niobium Nitride (NbN)

NbN is one of the alternative superconductors to Nb for SRF applications. NbN T_c_ (~16 K) is higher than Nb, which allows for higher-temperature operation, and it has a greater chemical stability, making it resistant to surface oxidation. NbN is also a potentially interesting option for SIS multilayers, although bulk NbN intrinsic H_c1_ (~20 mT) is less than that of bulk Nb.

**Figure 13:**
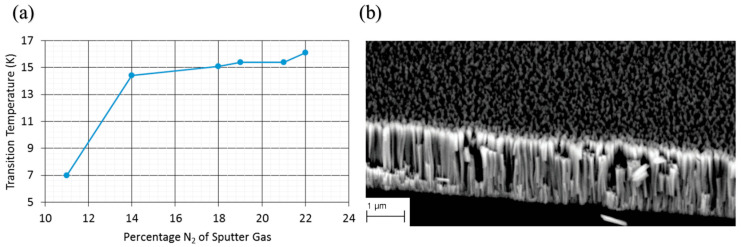
(**a**) Transition temperature of NbN thin films as a function of N_2_ partial pressure (substrate at 385 °C). (**b**) SEM cross-section of an NbN thin film (385 °C, 22% N_2_). Reproduced from [79].

Earlier studies confirmed the feasibility of NbN coating on cavities, but they also revealed the intrinsic limitations of these coatings. Reactive sputtered NbN films deposited at 510–580 °C on Nb substrates exhibited T_c_ of 16.2–16.5 K, H_c1_ ≥ 10 mT, and R_s_ ≈ 6.4–6.9 µΩ along with improved chemical stability and lower sensitivity to trapped flux as compared to Nb. However, their RF performance was limited by columnar microstructures and the intrinsically low thermal conductivity of NbN [77]. Multilayer concepts were developed to overcome these limitations. NbN films sputtered on Si substrates, and on Nb-SiO_2_-Nb multilayers on Nb plates, displayed a T_c_ of ~14.4 K. The multilayers achieved H_c1_ ≈ 220–230 mT at 2 K, exceeding the bulk Nb limit (~180 mT). This was the first experimental confirmation that NbN-based S-I-S multilayers can exceed the H_c1_ limit of Nb, which confirms the theoretical prediction [78]. Given NbN was established as a viable superconductor for S–I–S multilayers, optimisation of microstructure and NbN stoichiometry became the research focus. The HiPIMS deposition of NbN on Si(100) using Kr–N_2_ plasmas showed a very high sensitivity to nitrogen content. At 385 °C, T_c_ increased with increasing N_2_ partial pressure to a maximum of 16.1 K at ~22% N2 (Figure 13a). Even with this improvement, the films exhibited a columnar morphology with voids and relatively high resistivity (~835 ± 260 µΩ·cm), evident in Figure 13b. Film depositions performed at room temperature displayed an even lower T_c_ (10.3 K at 22% N_2_) with significantly higher resistivity (~2094 ± 650 µΩ·cm), indicating that elevated temperatures are essential for adequate mobility of the adatoms to ensure dense growth [79].

**Figure 14:**
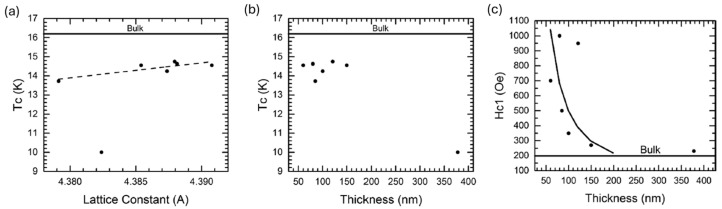
Variation in superconducting properties in NbN thin films: (**a**) T_c_ vs lattice constant, (**b**) T_c_ vs thickness, and (**c**) H_c1_ vs thickness (the thick line represents the calculated H_c1_ enhancement for NbN thin films assuming a coherence length of 4 nm). Reproduced from [81].

Complementary studies showed that the superconducting properties of NbN are very sensitive to stoichiometry. NbN films were deposited at room temperature by reactive DCMS on SiO_2_ with an N_2_ fraction of 0.5–30%, and a 50 V rf substrate bias. The films exhibited nearly stoichiometric δ-NbN (R_N2_ = 8–16%) with a T_c_ ≈ 12.8–13.2 K and a H_c2_ (0) ≈ 28–30 T. N-deficient films (1–2%N_2_) exhibited highly suppressed T_c_ ≈ 2.5 K, while N-rich films (≥20% N_2_) exhibited degraded T_c_ ≈ 6–7 K. The films were non-superconducting at ~30% N_2_. Both deficiency and excess N_2_ caused higher resistivity and lowered T_c_. This implies that good superconducting properties at room temperature, as well as elsewhere, require controlled stoichiometry [80]. To determine if NbN could serve as a direct coating material for SRF cavities, Leith et al. deposited NbN on polycrystalline Cu substrates using reactive DCMS and evaluated different pressures and N_2_ flow rates. At 6 × 10^−3^ mbar and a N_2_ fraction of ~8%, a δ-NbN (200) phase was obtained with a T_c_ ≈ 12 K and H_en_ ≈ 13 mT, and the microstructure was dense, with no apparent voids. At 1.4 × 10^−2^ mbar, δ-NbN(111) was the predominant phase with a high T_c_ of up to 16.1 K, but low H_en_ ≈ 5.0 mT due to the formation of faceted and discontinuous growth. Excess N_2_ (>15) caused secondary phases, severely reducing both T_c_ and H_en_ [68]. Thus, there is a clear trade-off, as higher T_c_ can be attained, but at the expense of the effectiveness of magnetic screening (H_en_) from the porosity or other secondary phases. The impact of lattice parameters and film thickness on superconducting properties was studied by depositing epitaxial NbN films on MgO(100) via DCMS. A linear correlation was identified between T_c_ and lattice parameters, shown in Figure 14a. There was no noticeable dependence of T_c_ on thickness (Figure 14b). H_c1_ has a significant thickness dependence, ~100 mT for an 80 nm film compared to ~20 mT for bulk NbN (Figure 14c). Therefore, it is necessary to control the sub-penetration-depth thickness for multilayer applications [81].

Based on these results, Burton et al. showed strong field enhancements in multilayers. Nb/MgO/NbN trilayers with ~85 nm top layers of NbN exhibited T_c_ ≈ 13 K, and H_c1_ ≈ 210 mT; confirming that NbN multilayers are excellent candidate materials for SIS screening in SRF cavities [25]. In summary, NbN has been studied mainly within multilayer architectures, although standalone NbN coatings on Cu have also been investigated. Control of the stoichiometry and thickness has been shown to lead to T_c_ values up to ~16 K and moderate H_en_. However, the use of NbN in SIS multilayers is the most viable application, where thin, controlled NbN layers can help to extend Nb cavity performance beyond its intrinsic limits.

### 5.3. Niobium Titanium Nitride (NbTiN)

Alloying NbN with Ti has been investigated as a way to enhance the properties of nitride-based superconductors for SRF applications. NbTiN, compared to NbN, has lower normal-state resistivity, smoother microstructures, and higher T_c_ values, while maintaining the chemical stability of nitrides. These properties make NbTiN an attractive compound for applications either as a direct coating or as the outer superconducting layer in SIS multilayers.

**Figure 15:**
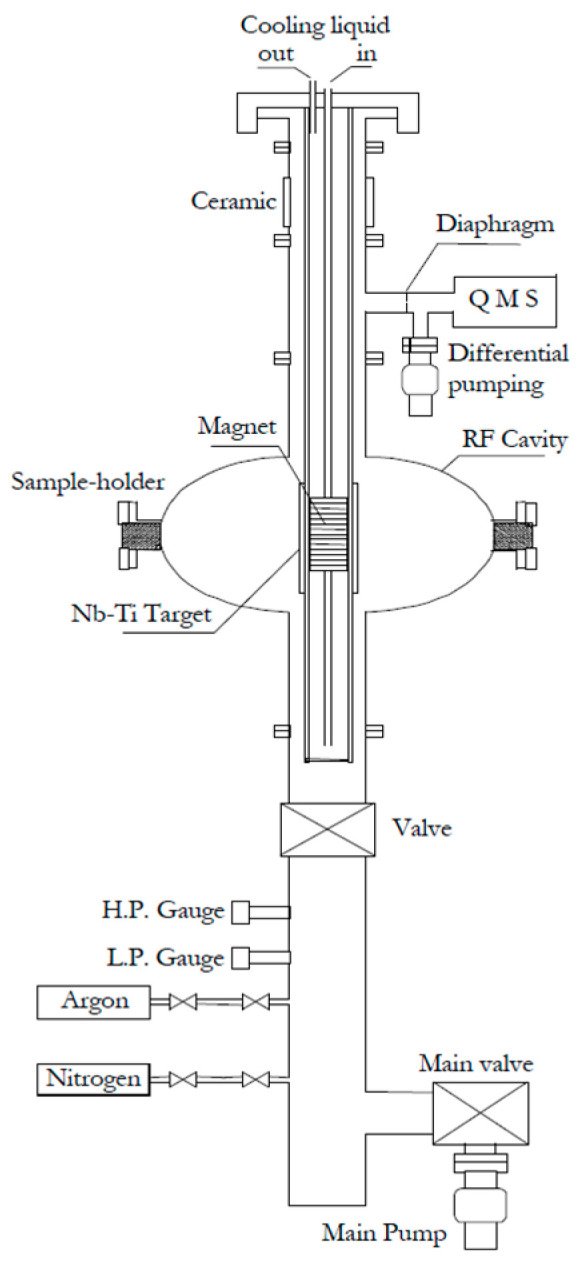
The sputtering system for the deposition of NbTiN thin films inside 1.5 GHz resonators. Reproduced from [84].

The first experiments have shown that Ti alloying of NbN will significantly reduce resistivity without degrading superconductivity. Films deposited by reactive DCMS at 200–600 °C on sapphire substrates have achieved T_c_ values greater than 16.8 K and exhibited a relatively sharp transition (ΔT_c_ ≤ 0.1 K). With regard to NbN (ρ ≈ 170 µΩ·cm, R_s_ ≈ 5.4 nΩ at 200 °C), the addition of Ti decreased the resistivity to ~62 µΩ·cm and R_s_ to ~2.1 nΩ without affecting T_c_. All of these improvements can be achieved at significantly lower deposition temperatures, positioning NbTiN as a more attractive option than pure NbN for SRF applications [82]. Expanding on this work, additional studies were conducted to investigate RF performance relevant to cavity behaviour. Optimised (Nb_0.55_Ti_0.45_)N coatings on Cu and SiO_2_ substrates achieved T_c_ ≈ 16.0–16.3 K with relatively sharp transitions (ΔT_c_ ≈ 0.1 K). RF tests in a 4 GHz cavity showed residual surface resistance as low as 40 nΩ at 1.6 K, comparable to bulk Nb; however, when the cavity was tested at 4.2 K, the R_s_ was up to 5 times lower than that of Nb. A maximum RF field of 34 mT (E_acc_ ≈ 8.5 MV/m) was achieved before quench, which confirms the potential of NbTiN films for higher-temperature SRF operation [83]. Cylindrical magnetron methods were subsequently developed to extend these results to geometries at the cavity scale. Nb_0.35_Ti_0.65_ films were deposited inside 1.5 GHz seamless Cu cavities using a Nb–Ti central cathode, which is adapted from the CERN Nb/Cu setup (Figure 15). Optimised films on quartz achieved T_c_ ≈ 15.5 K, RRR ≈ 1.45, and ρ_0_ ≈ 35 µΩ·cm. Coatings on Cu cavities displayed T_c_ ≈ 14.2 K, residual resistance ~350 nΩ, and a BCS resistance of 55 nΩ at 4.2 K, which is significantly lower than Nb/Cu (~400 nΩ) and bulk Nb (~900 nΩ) at the same frequency, although residual resistance remained high. These results established NbTiN as one of the first non-Nb films realistically tested in SRF cavities [84].

After demonstrating the feasibility of the cavity, optimisation turned to controlling deposition temperature, buffer layers and substrate. Films deposited on oxidised Si at 450 °C exhibited T_c_ > 16 K, smooth surfaces (rms ≈ 1.5 nm) and penetration depths of 200 ± 20 nm at 10 K, with coherence length similar to that of epitaxial NbN. Superconducting properties deteriorated at low temperatures, although ~0.5 K improvement in T_c_ is observed for a 40 nm Nb buffer layer [85]. Substrate material was also proven to be critical to obtain high T_c_ films. In a study [86], NbTiN films deposited on MgO(100) at 600 °C exhibited T_c_ ≈ 16.2 K, whereas deposition on AlN or Al_2_O_3_ resulted in slightly lower T_c_ values (14–15 K). Alongside such optimisations, advanced sputtering techniques were employed to further improve film quality. Using dual magnetron sputtering (Nb powered by HiPIMS, Ti using pulsed DC) in Kr/N_2_ atmospheres. superconducting films were achieved for N_2_ partial pressures ≥ 14%, with the highest T_c_ of 17.8 K at 20% N_2_. The respective films showed dense, fine-grained fcc (111) structures, with a normal-state resistivity of 45 ± 7 µΩ·cm; which is nearly an order of magnitude lower than the best NbN films produced by HiPIMS and other methods under comparable conditions. This provides evidence that HiPIMS can produce NbTiN films with high T_c_ and significantly enhanced electrical performance [79].

In addition to the above improvements, studies were carried out systematically to evaluate the effect of alloy composition and nitrogen content. At 2.5% N_2_, epitaxial NbTiN grown from Nb/Ti alloy targets, at approximately 600 °C, resulted in B1-phase structures with lattice parameters of 4.30–4.36 Å. The films produced from both the 80/20 (wt.%) Nb/Ti targets exhibited T_c_ values of 16.7 K and H_en_ on the order of 200 mT, whereas the films produced from the 70/30 (wt.%) targets achieved H_en_ of ≤83 mT. Therefore, it can be concluded that greater stoichiometry and crystallinity enhance T_c_ and provide stronger flux screening [87].

In another work, ultrathin limits were investigated: optimised 300 nm films deposited on SiO_2_/Si exhibited a T_c_ of ~15.5 K (ΔT_c_ ≈ 0.03 K) and ultralow surface roughness (less than 0.2 nm). Even 5 nm thin films exhibited superconductivity (T_c_ ≈ 7.6 K). Rapid thermal annealing in N_2_/H_2_ atmospheres increased the T_c_ of 10 nm films from 9.6 K to 10.3 K by grain coarsening and texture reorientation from [111] to [100] [88]. In a recent study, nitrogen control was found to be equally decisive. The N_2_ partial pressure was varied from 5.8% to 15.15% relative to the Ar flow. At 600 °C, epitaxial films with a thickness of 50 nm on MgO(001) exhibited the best performance at N_2_ = 6.8% N_2_, with a T_c_ of 15.77 K (ΔT_c_ ≈ 0.14 K) and the (200)/(400) orientations. The behaviour of T_c_ as a function of nitrogen content was found to be nonlinear, while Bc(0), the diffusion coefficient, and the coherence length were linear. In contrast, films deposited at room temperature were amorphous, and they exhibited suppressed T_c_ (~10–11 K). This study indicates the importance of a narrow nitrogen window (5.8–8.5%) for obtaining high-quality NbTiN [89].

In conclusion, NbTiN has moved from thin film demonstrations to coatings on cavity scales to SIS multilayer integration. Optimised deposition, especially with HiPIMS-assisted yield T_c_ as high as 17.8 K with dense, low-resistivity films. While there are still many issues to address with residual resistance, stress defects, and nitrogen stoichiometry, NbTiN is one of the most exciting nitride superconductors for future SRF coatings.

### 5.4. Niobium Tin (Nb_3_Sn)

Nb_3_Sn, with a T_c_ of ~18.3 K and H_c1_ ≈ 50–70 mT, is the most extensively investigated A15 superconductor for SRF cavities. Nb_3_Sn allows more efficient operation at 4.2 K compared to Nb, providing a significant reduction in cryogenic costs. Conventional Nb_3_Sn coatings are produced by Sn vapour diffusion, but sputtering can provide tunable control over composition, thickness and microstructure, and could be deposited on copper substrates.

**Figure 16:**
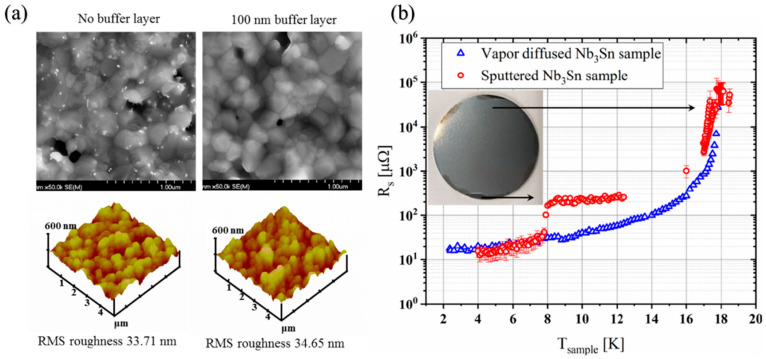
(**a**) SEM and AFM images of Nb_3_Sn films with and without a 100 nm Nb buffer layer, (**b**) Temperature dependence of surface resistance Rs for sputtered Nb_3_Sn compared with a vapour-diffused sample; inset shows a sputtered sample. Reproduced from [91].

Initial efforts used multilayer sputtering followed by annealing to form Nb_3_Sn. Nb/Sn multilayers were deposited at 3 mTorr using a Nb (DC) and Sn (RF) target and subsequently annealed at 1200 °C for 3 h, resulting in crystalline, uniform, void-free Nb_3_Sn with T_c_ as high as 17.6 K. Phase formation was closely correlated with the Nb-to-Sn layer thickness ratios with thinner stacks (~2) producing phase pure Nb_3_Sn layers while thicker stacks (~4.5) produced secondary Nb_6_Sn_5_ phases. Sn deficiency after annealing has remained an issue [90]. The multilayer strategy was further optimised by the addition of Nb buffer layers (0 to 100 nm) below stacks of Nb (20 nm) and Sn (10 nm) (total ~1.5 µm). After annealing at 950 °C for 3 h, uniform grain growth was observed, with surface roughness between 23 and 34 nm (Figure 16a). All coatings exhibited T_c_ ≈ 17.75–17.82 K with narrow transitions, of RRR ≈ 4.3–4.7, and surface resistances similar to films from vapour-diffused films (Figure 16b) [91].

**Figure 17:**
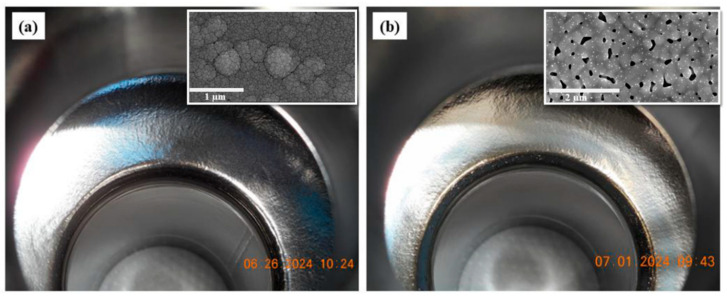
Surface of the coated Nb cavity: (**a**) as deposited and (**b**) after sequential annealing at 600 °C for 6 h followed by 950 °C for 1 h. Insets show corresponding SEM micrographs of the surface morphology. Reproduced from [94].

The possibility of using co-sputtering of Nb and Sn onto sapphire substrates was investigated to simplify the process. The crystallinity and T_c_ of the deposited films at substrate temperatures of room temperature (RT) to 500 °C were dependent on the growth and annealing conditions. Films deposited at 500 °C that were not annealed exhibited a T_c_ of ~15.0 K, while RT films, after annealing at 665 °C and 950 °C, displayed T_c_ ≈ 15.9 K and T_c_ ≈ 17.6 K, respectively. This confirmed that co-sputtering and annealing at higher temperatures produce high-quality Nb_3_Sn suitable for SRF cavities [92]. The effects of annealing and film thickness on the formation and stability of Nb_3_Sn coatings were investigated in more detail. Films of thicknesses 100 nm, 300 nm, and 2 µm were deposited by DCMS from a stoichiometric Nb_3_Sn target onto Nb and Cu substrates. After a 6-h anneal at 600–950 ˚C, thin 100 nm layers on Nb recrystallised well, with T_c_ ≈ 17.5 K, while thicker 2 µm films lost Sn or did not completely form Nb_3_Sn, and Cu films decomposed. The findings highlighted that thinner Nb_3_Sn on Nb substrates are more stable, whereas thicker films and coatings on Cu are less stable [93]. In working towards cavity applications, Shakel et al. developed a co-sputtering process, designed specifically for cylindrical geometries. In this work, using a cylindrical magnetron system, Nb–Sn was sputtered with 32–42 at.% Sn, which crystallized into Nb_3_Sn after annealing at 950 °C. As shown in Figure 17, a 1.5 µm thick film was successfully deposited on a 2.6 GHz Nb cavity and annealed in two steps (600 °C/6 h and 950 °C/1 h). The resulting coating exhibited T_c_ ≈ 17.8 K. A light Sn recoating further improved stoichiometry and uniformity, which enhanced RF performance to Q_0_ ≈ 8.5 × 10^8^ at 2.0 K. This study demonstrated the viability of sputtered Nb_3_Sn for cavity integration [94].

Another attempt was made on Cu substrates, where Nb_3_Sn films of 1.5–2 µm thickness were deposited using DCMS from stoichiometric Nb–Sn targets in Ar or Kr atmospheres. The process gas strongly influenced composition, with Kr increasing the Sn content by ~3 at.% compared to Ar. However, the best performing coatings based on Cu reached T_c_ ≈ 16 K, interdiffusion between the film and the substrate suppressed T_c_ by around 2–3 K compared to Nb substrates. The extended annealing to ~750 °C for 24 h improved the ordering of the films and raised T_c_ [95].

In summary, sputtered Nb_3_Sn films have achieved T_c_ values of up to ~17.8 K with microstructures comparable to those of vapour-diffused coatings. The results of these studies indicate that sputtering allows flexibility to control stoichiometry and microstructure. However, Sn deficiency, interdiffusion with Cu, and even strain in thick coatings are still causing reproducibility and stability issues. Therefore, optimisation of deposition and annealing is required before sputtered Nb_3_Sn can be considered as an alternative to vapour diffusion in real-world applications of SRF cavities.

### 5.5. Niobium Aluminide (Nb_3_Al)

Nb_3_Al as the A15 intermetallic superconductor, with a T_c_ of almost 18 K and an H_c2_ of over 20 T, is very suitable for SRF applications. It has better mechanical toughness and enhanced irradiation resistance than Nb_3_Sn, which can help relieve thermal and mechanical stress in the SRF cavity. While there are distinct advantages with Nb_3_Al, the existing research on this material has primarily focused on electronic and device applications with little attention given to its potential for SRF cavities.

Thin films of Nb_3_Al were first made by magnetron sputtering from arc-melted targets, and reported the highest T_c_ values for as-sputtered films (up to 16.7 K). The film properties showed a strong dependence on the deposition parameters: well-ordered A15 Nb_3_Al with a small amount of Nb_2_Al deposited at 650–700 °C exhibited T_c_ values above 16 K, but lower temperature deposits displayed disordered structures and low T_c_ values. Argon pressure also showed a significant influence, as applying less than 10 Pa induced a systematic decrease in T_c_, indicating that sufficient thermalisation and reduced substrate bombardment are required to obtain high-T_c_ Nb_3_Al films [96]. In later studies, single-phase Nb_3_Al targets produced from melt-and-cast processing were used for RF magnetron sputtering. Films deposited on sapphire substrates (after annealing the films at ~865 °C) yielded T_c_ ≈ 13.8 K, while films on MgO substrates exhibited relatively lower T_c_. A15 phase was confirmed by structural analysis, and it was demonstrated that substrate choice significantly affects T_c_, and sapphire allows for more interfacial ordering than MgO [97]. More recent work introduced DC co-sputtering from separate Nb and Al targets. As-deposited films at 700–830 °C were severely disordered with a T_c_ of ~12 K due to aluminium segregation and poor nucleation. Rapid thermal annealing at near 1000 °C, improved the film crystallinity and produced a T_c_ as high as ~15.7 K for ~300 nm films with ~24 at.% Al. The use of a Nb seed layer improved the texture and crystallographic order, which resulted in improved T_c_ ≈ 15.3 K with sharper diffraction peaks. The superconducting parameters H_c2_ (0) ≈ 17.8 T, ξ ≈ 4.3 nm, and λ ≈ 210 nm were also obtained, which are comparable to NbN and NbTiN thin films [98].

In summary, sputtered Nb_3_Al films have shown competitive superconducting properties with T_c_ values above 16 K in optimised conditions. However, they are extremely sensitive to stoichiometry, substrate choice, annealing, and secondary phases. Nb_3_Al is attractive for many reasons, including its high T_c_, large H_c2_, and mechanical strength; however, there has been no systematic investigation for SRF cavity coatings. This is an under-utilised opportunity for developing SRF thin-film technology.

### 5.6. Vanadium Silicide (V_3_Si)

V_3_Si is an A15-type superconductor with a bulk T_c_ approaching ~17 K and an H_c2_ of over 20 T; therefore, it remains an interesting candidate for SRF. Like the other A15 compounds, V_3_Si is brittle, creating difficulties when considering its use in bulk applications. However, thin-film deposition through sputtering may allow us to take advantage of its favourable superconducting properties.

Early studies focused on post-deposition annealing of sputtered V films. Upon annealing on silica-based substrates at temperatures between 300–900 °C, the superconducting properties were significantly influenced by temperature and substrate. In the case of deposition on Vycor substrate, T_c_ was observed to be <1.4 K after annealing at temperatures < 600 °C, but it rose to ~12 K at this threshold and finally rose to ~13 K at 750 °C, prior to some partial flaking occurring at ~900 °C. On quartz, T_c_ values of 11.1–13.3 K were obtained with an anneal at 725 °C; while on Si, an anneal at 900 °C led to T_c_ values up to 14.7–16.8 K, gaining bulk-like performance. A film annealed at 800 °C on Vycor had the best upper critical field H_c2_ (4.2 K) ≈ 13–17.5 T, which confirms that one can get high-quality V_3_Si films despite the inherent brittleness of the compound [99]. To overcome substrate constraints and enhance compositional control, V_3_Si was grown on sapphire by reactive DCMS from V targets in Ar–SiH_4_ mixtures at ~15 mTorr. Films grown at temperatures above 700 °C obtained the best properties, with T_c_ ≈ 16.8 K, while V-rich films (with >75 at.% V) exhibited lower T_c_ (~15 K) due to inhomogeneity from grain-to-grain. While the A15 phase may be able to form below 300 °C, the limited value of T_c_ (7–9 K) shows both the sensitivity of T_c_ to growth conditions and the inherent difficulties of achieving stoichiometric uniformity in reactively sputtered V_3_Si [100]. The following work used stoichiometric V_3_Si targets and direct sputtering to produce uniform films on substrates relevant to SRF. Films were deposited on Cu and sapphire using pulsed DCMS in Kr at 790 °C with an average thickness of ~1.4 μm. The coatings had a slight Si excess (V:Si ≈ 72:28). All films exhibited a granular structure; films on Cu had small, dense grains relative to those on sapphire. The two samples exhibited superconductivity with T_c_ ≈ 14.3 K on sapphire and 12.8 K on Cu. The magnetic field at first penetration was B_fp_ (4.2 K) = 61 mT for sapphire, and B_fp_ (4.2 K) = 19 mT for Cu. Sapphire performed relatively better than Cu, showing the detrimental effects of Cu interdiffusion [101].

**Figure 18:**
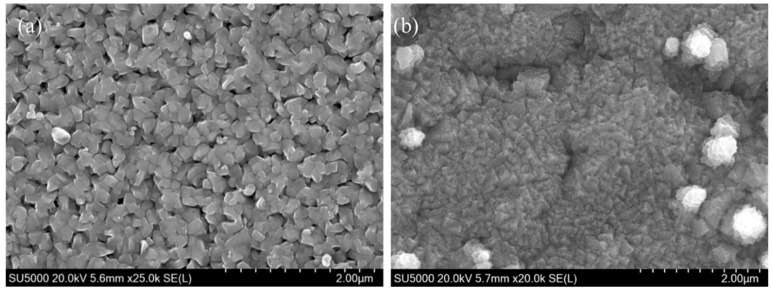
SEM images of V_3_Si films deposited by HiPIMS on (**a**) a sapphire substrate and (**b**) a Nb substrate. Reproduced from [102].

More recently, high-ionisation techniques such as HiPIMS have been employed to improve density and microstructure. V_3_Si films deposited from stoichiometric targets in Kr (3 × 10^−3^ mbar, 300 W, 1 kHz, 10 µs pulse, 10% duty cycle) exhibited contrasting morphologies depending on the substrate. The sapphire coatings were porously large-grained, while the coatings on Nb had finer crystallites, which were affected by the rougher surface of the substrates (Figure 18a,b). The T_c_ values were 14.85 K and 13 K for sapphire and Nb, respectively, and both were below the bulk limit, which is attributed to small grain size and contamination during growth [102].

Further studies added to the understanding of the importance of annealing to produce the A15 phase and relieve strain. Films deposited by DCMS on Nb and Cu substrates and annealed between 600–950 °C exhibited different behaviour. The 2 μm layers on Nb initially had approximately 15% strain, which was relieved during annealing in the range of 800–950 °C, enabling a phase transition to the stable V_3_Si phase. On Cu, the 300 nm films contained Cu–Si phases due to interdiffusion at 550 °C. An anneal at 950 °C removed the Cu–Si phases but left Cu inclusions and surface-like artefacts that degraded performance in the films. Observation of this study here shows that annealing stabilises V_3_Si and significantly removes strain in the film on Nb, while noting that Cu substrates require buffer layers for SRF coatings to mitigate interdiffusion [93].

Overall, sputtered V_3_Si films have demonstrated T_c_ values up to ~16.8 K and H_c2_ exceeding 17 T, showing promise for SRF applications. However, there are still many challenges, including difficulty with stoichiometry control, interdiffusion and inclusion that can arise from substrate interactions. For V_3_Si to develop as a potential alternative superconductor for SRF cavity coatings, these issues need to be overcome with optimised annealing, buffer-layer engineering, and advanced sputtering methods.

### 5.7. Mo-Re Alloys

Mo-Re alloys have been investigated as SRF coating materials due to the combination of relatively high T_c_ with low impurity solubility and desirable RF properties [23]. The ability to stabilise metastable A15 phases in thin films also contributes to their suitability for cavity applications [103].

The first stabilisation of an A15 Mo–Re phase in thin films was achieved via sputtering from composite Mo–Re targets at ~500 °C in ultra-high vacuum. A ~6 μm thick film deposited on sapphire showed superconducting T_c_ as high as ~15 K, and the transitions were very sharp (ΔT_c_ ≤ 0.5 K). The transitions occurred at ~9 K for Mo_0.3_Re_0.7_ (tetragonal phase) and ~13–15 K for Mo_0.7_Re_0.3_ (A15 phase), suggesting that phase composition is directly related to T_c_. The initial signs of A15 phase stabilisation prompted further studies that sought to optimise deposition temperature for tuning superconducting and RF properties [103]. Andreone et al. used DC magnetron sputtering to deposit Mo_75_Re_25_ films on sapphire at 1 × 10^−3^ Torr Ar pressure and substrate temperatures ranging from 100 °C to 900 °C. The films exhibited stoichiometric A15 phase, with T_c_ ≈ 9–10.3 K, and RRR increased from 1.4 at 100 °C to 2.5 at 900 °C. The calculated BCS surface resistance at 500 MHz and 4.2 K reached a value as low as 10 nΩ, which is still much lower than that of Nb (40 nΩ) [104]. The effects of composition and annealing were then studied in a systematic way. Films of compositions Mo_75_Re_25_, Mo_60_Re_40_, and Mo_38_Re_62_ were deposited by magnetron sputtering at 600–1000 °C and annealed in situ. For Mo_75_Re_25_, the highest values of T_c_ ≈ 11.82 K and exceptionally low ΔT_c_ = 0.012 K were produced by annealing in the range of 751–793 °C (RRR = 1.71). The Mo_60_Re_40_ films, annealed at a temperature range between 800–856 °C, produced the highest T_c_ = 12.13 K (ΔT_c_ = 0.065 K, RRR = 1.3), while the Mo_38_Re_6_ films exhibited T_c_ ≈ 9.47 K. In every case, the improvement in homogeneity, transition sharpness, and T_c_ was clearly seen across the composition range studied. BCS surface resistance at 500 MHz and 4.2 K was ~16 nΩ for Mo_60_Re_40_ and Mo_75_Re_25_, which was significantly less than sputtered Nb (~64 nΩ), suggesting that the films to be potentially useful for SRF use [105].

**Figure 19:**
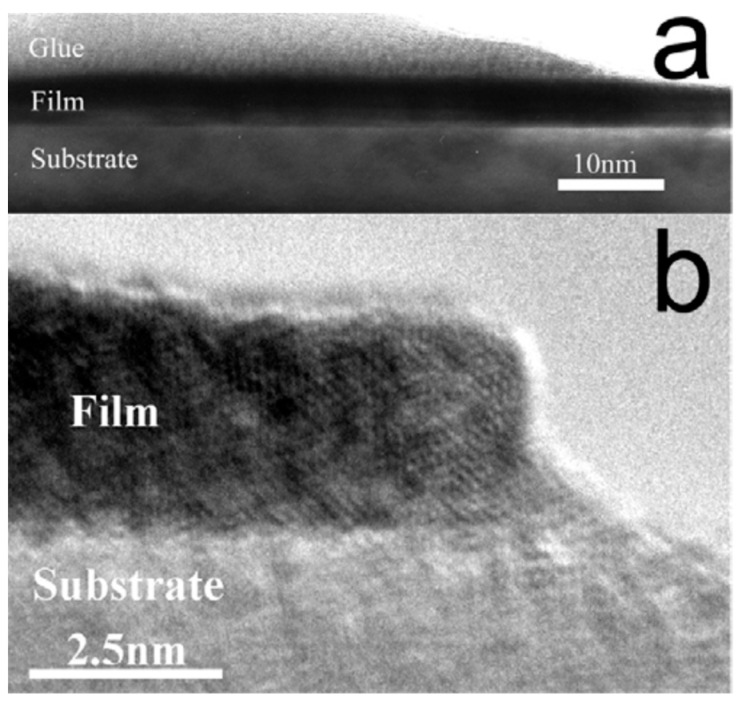
Cross-sectional HRTEM image of a 4 nm Mo_60_Re_40_ film (**a**) Low-magnification view showing the continuous film, substrate, and glue layer. (**b**) High-resolution view of the film–substrate interface, confirming a homogeneous structure without breaks or exfoliations. Reproduced from [106].

With these studies being mostly focused on bulk-like thick films, subsequent investigations began to focus more on ultrathin Mo–Re layers. Seleznev et al. fabricated 2–10 nm Mo_60_Re_40_ films on sapphire by DCMS at 3 × 10^−3^ Torr Ar and with substrate temperatures ranging from 150–600 °C. The films displayed uniform structure, and both stoichiometric transfer was achieved (Mo/Re ≈ 0.575/0.425), albeit with ~10% oxygen contamination. The superconducting temperature was sensitive to thickness and deposition conditions: T_c_ = 4.2–5.2 K (2 nm), 5.2–7.7 K (4 nm), and 9.7 K (10 nm) with narrow transitions (ΔT_c_ = 0.1–0.3 K). Increasing the substrate temperature improved the film quality, and consequently, the T_c_ increased 4.75 K at 170 °C to 7.0 K at 380 °C, and then dropped to 6.2 K at 600 °C. HRTEM images indicated that the films possess continuous and homogeneous microstructure throughout the thickness of the films, without any breaks or exfoliation (Figure 19a,b) [106].

Overall, sputtered Mo–Re films show excellent superconducting and RF properties, including T_c_ ≈ 15 K in thick films and ~10 K in ultrathin layers, and calculated surface resistances are much lower than Nb, emphasising their use for SRF. Further understanding of controlling stoichiometry, thickness, and impurity incorporation (especially oxygen), will be key to unlocking the potential of Mo–Re as an SRF coating material, both in bulk-like films and multilayer cavity concepts.

### 5.8. Magnesium Diboride (MgB_2_)

MgB_2_ was discovered in 2001, with a T_c_ of ~39 K [107]. The crystal structure of MgB_2_ is hexagonal, with all boron layers in a closed-packed arrangement separated by the magnesium layers. Unlike the majority of superconductors, MgB_2_ shows a novel two-gap behaviour with Δπ ≈ 2.7 meV and Δσ ≈ 6.7 meV [108]. It is a promising candidate for SRF cavities in the 15–20 K range because of its relatively high T_c_, moderate H_c2_, and extremely low resistivity in the normal state (≤1 μΩ·cm) [23]. However, the application of MgB_2_ thin films for SRF cavities is limited by several drawbacks, including the high volatility of Mg, difficulties in depositing dense and stoichiometric films, and a very high sensitivity to oxygen contamination [109].

**Figure 20:**
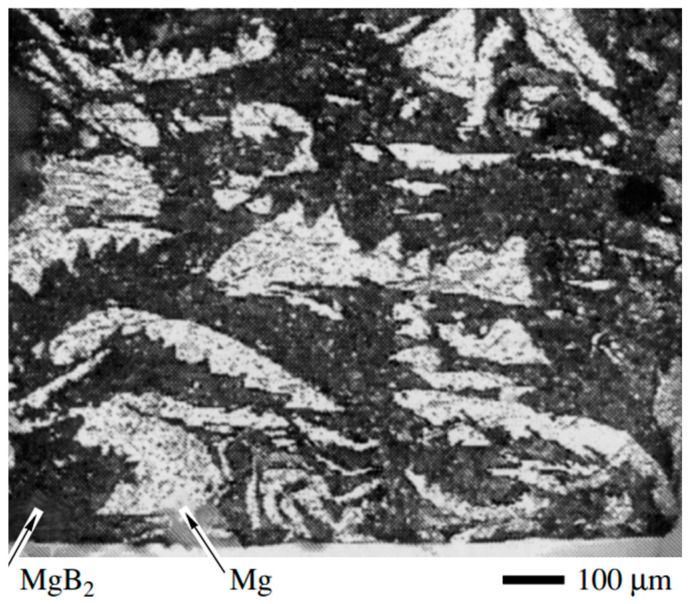
Cross-sectional fragment of the composite Mg–MgB_2_target, where the black regions correspond to MgB_2_ and the white areas to metallic Mg. Reproduced from [107].

In the first demonstration of MgB_2_ thin films using DCMS, a composite MgB_2_–Mg (∅32 mm) target was used. The target was particularly made to mitigate losses of Mg during the oxidation of plasma. The dense structure of the target enabled good heat transfer, stable sputtering, and prevented target cracking even at higher power densities. A cross-sectional fragment of the composite target (Figure 20) shows that the black regions correspond to the MgB_2_ and the white areas to metallic Mg. Films were deposited on sapphire with high-purity Ar (3 Pa, ~120 W/cm^2^) at 200 °C and were followed by a short in situ annealing at 600 °C. The obtained films were fine-grained and uniform in morphology with T_c_ ≈ 24 K. Although the morphology was promising, the measured resistivity was over two orders of magnitude, greater than that of bulk MgB_2_, which is attributed to the small grain size [107].

**Figure 21:**
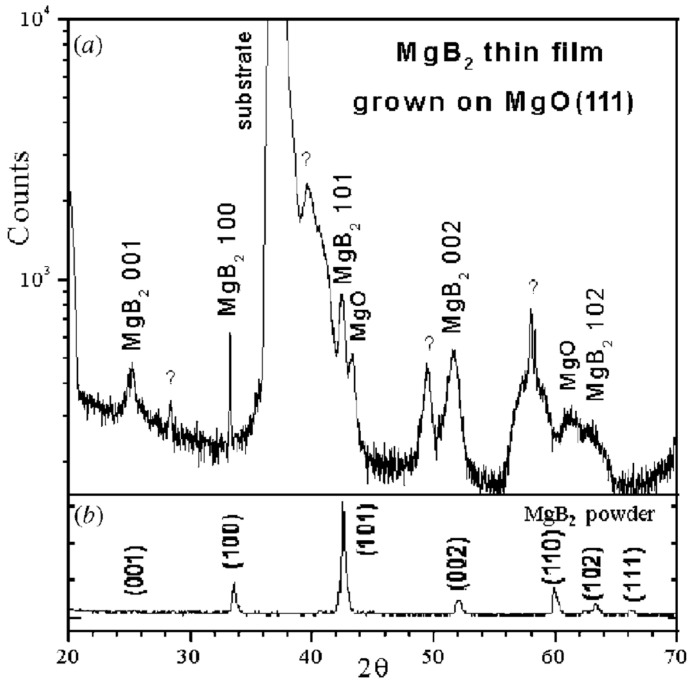
(**a**) XRD spectrum of a MgB_2_ film deposited on a MgO(111) substrate, showing MgB_2_ phase formation with minor MgO and unidentified phases. (**b**) Reference XRD pattern of MgB_2_ powder for comparison. Adapted from Vaglio et al. [7].

While this pioneering work demonstrated the feasibility of sputtering MgB_2_, subsequent attempts quickly revealed how severe Mg volatility can be in destabilising film stoichiometry. MgB_2_ thin films were sputtered from compacted powder targets (3 mm) at 4 × 10^−3^ mbar Ar, 5 sccm flow, using 25 W pulsed DC power for 4 h. The films were dense, pore-free, and adherent; however, the Mg/B atomic ratio was lower than the desired 1:2 stoichiometry, which is explained by the high volatility of Mg relative to B. As a result, no superconductivity was observed. It was suggested that this limitation could be overcome by introducing a Mg overpressure during deposition and by using lower sputtering powers to reduce heat generation and hence the rate of Mg vaporisation [110]. Recognising these limitations, other groups studied co-deposition routes combined with post-annealing to control stoichiometry. Mičunek et al. prepared MgB_2_ thin films on sapphire substrates by co-deposition of boron (RF magnetron, 250 W) and magnesium (DC magnetron, 29 W) at a working Ar pressure of 7.4 × 10^−2^ Pa, directly on unheated sapphire substrates, followed by ex situ annealing in Ar. Following 40 min of deposition, a ~200 nm Mg-B precursor film was produced, which was then annealed at 620–680 °C. The best results were obtained at 680 °C for 2.5 min, where the films showed an onset T_c_ of ≤35 K. However, Auger analysis indicated that excess magnesium and contamination from oxygen decreased the performance of the film [111]. With the development of Mg vapour-assisted sputtering, a major milestone was achieved with regard to film quality and reproducibility. In this method, a two-step in situ DCMS process was used to deposit Mg–B precursor layers in high-purity Ar (9 × 10^−1^ Pa) from MgB_2_ and Mg targets, followed by annealing at 830 °C for 10 min inside a sealed Nb box under Mg vapour. This novel approach provided a saturated Mg vapour environment that improved the film quality and reproducibility. The XRD pattern (Figure 21) for a film deposited on crystalline MgO (111) confirmed the MgB_2_ phase with small amounts of MgO (from oxidation of excess Mg) and minor unidentified phases. AFM showed a granular surface with columnar grains a few microns wide and ~50 nm roughness. The films (~0.8–1 µm) on sapphire and MgO substrates exhibited T_c_ ≈ 35 K with a narrow ΔT_c_ ≈ 0.5 K, RRR ≈ 1.6, room-temperature resistivity ~200 µΩ·cm, and upper critical fields of H_c2⊥_ ≈ 2.9 T and H_c2‖_ ≈ 8 T at 25.7 K. This work represented one of the first demonstrations of high-quality in situ sputtered MgB_2_ films without ex situ annealing [7].

While improvements have been made, MgB_2_ films fabricated by sputtering remain considerably short of their bulk potential. Other deposition techniques, such as low-pressure CVD, pulsed laser deposition, and molecular beam epitaxy, produce higher quality MgB_2_ films, and sputtered MgB_2_ continues to have difficulties with challenges of Mg volatility, oxygen inclusion, and microstructural inhomogeneities. Further optimisation of Mg overpressure, substrate heating and contamination control is required before MgB_2_ can be a viable coating option for SRF cavities. If these challenges can be overcome, MgB_2_ may allow SRF operation at 15–20 K, an important milestone for cost-effective accelerators.

## 6. Conclusions and Outlook

Over the last few decades, sputtered Nb has demonstrated superior performance in SRF cavity applications, with accelerating gradients exceeding 20 MV/m and quality factors of ~10^9^. However, its performance remains limited by voids, impurities, non-uniformity, and adhesion issues. NbN and NbTiN have proven to be valuable candidates, particularly for SIS multilayer structures that have achieved superior magnetic screening and delayed flux penetration beyond that of bulk Nb. Among the A15 compounds, Nb_3_Sn thin films remain the most optimised; however, Sn deficiency and Cu interdiffusion remain challenging. Other materials, such as Nb_3_Al, V_3_Si, and Mo-Re, are still at an exploratory stage; MgB_2_ has the highest T_c_, allowing SRF operation to be performed at 15–20 K; nevertheless, it suffers from instability and high oxygen sensitivity. Overall, these studies show that while Nb remains the benchmark, sputtered nitrides, A15 compounds, and MgB_2_ provide key strategies for improving SRF thin-film technologies. Sputtering has established itself as the most versatile route for depositing superconducting thin films for SRF cavities. Across all material systems, dense, stoichiometric, uniform and adherent films have consistently higher T_c_, lower R_s_, and greater RF stability. Advances in the future can be made through three approaches: (i) energetic sputtering (i.e., HiPIMS) for dense homogeneous films, (ii) multilayer or interface engineering to exceed the fundamental limits of Nb, and (iii) systematic cavity demonstrations. If these advances are realised, sputtered films could become true high-performance SRF coatings for next-generation accelerators.

## Figures and Tables

**Table 1:** Comparison of superconducting material properties relevant for SRF cavities, Adapted from [23].

Material	T_c_ (K)	ρ_n_ (μΩcm)	H_c_ (0) (T)	H_c1_ (0) (T)	H_c2_ (0) (T)	λ (nm)	Δ (meV)	ξ (nm)
Nb	9.23	2	0.2	0.18	0.28	40	1.5	35
NbN	16.2	70	0.23	0.02	15	200–350	2.6	3–5
NbTiN	17.3	35	0.23	0.03	15	150–200	2.8	5
Nb_3_Sn	18	8–20	0.54	0.05	28	80–100	3.1	4
V3Si	17	4	0.72	0.072	24.5	179	2.5	3.5
Nb_3_Al	18.7	54			33	210	3	
MgB_2_	40	0.1–10	0.43	0.03	3.5–60	140	2.3/7.2	5

## Data Availability

No new data were created or analyzed in this study.
